# Real-world Persistence on Selexipag for Pulmonary Arterial Hypertension in Canada

**DOI:** 10.1016/j.cjco.2025.10.009

**Published:** 2025-10-25

**Authors:** Jason Weatherald, Steeve Provencher, Jillian Murray, Matthew Badin, Shane Golden, Lisa Mielniczuk, John Swiston, Chang Zhang, Cindy Y.Y. Yip, Jean-Claude Mamputu, Brad Millson

**Affiliations:** a Division of Pulmonary Medicine, Department of Medicine, University of Alberta, Edmonton, Alberta, Canada; b Institut universitaire de cardiologie et de pneumologie de Québec Research Center, Université Laval, Quebec City, Quebec, Canada; c IQVIA Solutions Canada Inc., Mississauga, Ontario, Canada; d Department of Medicine, Division of Cardiology, University of Ottawa Heart Institute and University of Ottawa, Ottawa, Ontario, Canada; e Department of Medicine, Division of Respirology, University of British Columbia, Vancouver, British Columbia, Canada; f Johnson & Johnson, Titusville, New Jersey, USA; g Johnson & Johnson, Toronto, Ontario, Canada

**Keywords:** selexipag, persistence, pulmonary arterial hypertension, Canada

## Abstract

**Background:**

Safety and tolerability of selexipag in pulmonary arterial hypertension (PAH) was demonstrated in a phase 3 clinical trial. In this trial, up to 14% of patients receiving selexipag over a median period of 70 weeks prematurely discontinued therapy due to adverse events. However, no data are available on real-world persistence on selexipag in Canada. This study aimed to describe selexipag short and long-term persistence, and predictors of persistence in the Canadian population.

**Methods:**

This retrospective claims-database analysis included adult PAH patients with ≥ 1 selexipag claim between April 2016 and July 2021. Patients were followed for a maximum of 64 months. Kaplan-Meier estimates of persistence were calculated from the index date to the earliest date of discontinuation or censoring. Four models were used to select predictors of persistence, and the 12 most important features based on average rank were built into a Cox proportional hazards model to assess the impact of these predictors on patient persistence with selexipag.

**Results:**

A total of 311 patients (70% female; 71% aged 50-79 years) were included in the study. The Kaplan-Meier estimates of persistence at 6, 12, 24, and 36 months were 76.1%, 61.7%, 48.3%, and 40.2% respectively. The median duration of persistence for the overall cohort was 22 months. No predictors in the final model were significant.

**Conclusions:**

Real-world data from the Canadian population suggest a low level of long-term persistence on selexipag, although the level of persistence is similar to reported rates for other PAH therapies. Further research into predictors of selexipag persistence is needed to help optimize potential treatment outcomes.

## Résumé

**Contexte:**

L'innocuité et la tolérabilité du sélexipag dans le traitement de l'hypertension artérielle pulmonaire (HTAP) ont été démontrées dans le cadre d'un essai clinique de phase III. Dans cet essai, jusqu'à 14 % des patients recevant du sélexipag sur une période médiane de 70 semaines ont interrompu prématurément le traitement en raison d'effets indésirables. Cependant, il n'existe aucune donnée sur la persistance thérapeutique réelle du sélexipag au Canada. Cette étude visait à décrire la persistance à court et à long terme du sélexipag, ainsi que les facteurs prédictifs de persistance dans la population canadienne.

**Méthodologie:**

Cette analyse rétrospective de bases de données des demandes de remboursement a porté sur des patients adultes atteints d'HTAP ayant présenté au moins une demande de remboursement pour du sélexipag entre avril 2016 et juillet 2021. Les patients ont été suivis pendant une période maximale de 64 mois. Les estimations du taux de persistance selon Kaplan-Meier ont été calculées à partir de la date de référence jusqu'à la date la plus proche d'arrêt ou de censure. Quatre modèles ont été utilisés pour sélectionner les facteurs prédictifs de la persistance, et les 12 caractéristiques les plus importantes selon leur classement moyen ont été intégrées dans un modèle de risques proportionnels de Cox afin d'évaluer l'impact de ces facteurs prédictifs sur la persistance des patients sous sélexipag.

**Résultats:**

Au total, 311 patients (70 % de femmes; 71 % âgés de 50 à 79 ans) ont été inclus dans l'étude. Les estimations de la persistance selon Kaplan-Meier à 6, 12, 24 et 36 mois étaient respectivement de 76,1 %, 61,7 %, 48,3 % et 40,2 %. La persistance médiane pour l'ensemble de la cohorte était de 22 mois. Aucun facteur prédictif n'était significatif dans le modèle final.

**Conclusions:**

Les données réelles issues de la population canadienne suggèrent une faible persistance à long terme du selexipag, bien que celle-ci soit similaire aux taux rapportés pour d'autres traitements de l'HTAP. Des recherches supplémentaires sur les facteurs prédictifs de la persistance du selexipag sont nécessaires afin d'optimiser les résultats potentiels du traitement.

Pulmonary arterial hypertension (PAH) is a pulmonary vascular disease characterized by progressive remodelling of the small pulmonary arteries.^1^ The most common symptoms of PAH are progressive breathlessness, clinical signs of heart failure, syncope, and fatigue.^2^ Estimates of the incidence and prevalence of PAH range from 1.5-32 per million and 12.4-268 per million, respectively.^3^

A number of Health Canada-approved treatments are available for PAH, including parenteral prostacyclin analogues, the oral prostacyclin receptor agonist selexipag, endothelin receptor antagonists (ERAs), phosphodiesterase 5 inhibitors (PDE5is), soluble guanylate cyclase stimulator riociguat, and the activin signaling inhibitor sotatercept.^4^^,^^5^ Selexipag was approved by Health Canada in 2016 for long-term treatment of PAH in adult patients with World Health Organization functional class II-III, to delay disease progression.^6^ In the phase 3 Prostacyclin (P**G**I2) **R**eceptor Agonist **I**n **P**ulmonary Arterial **H**ypertensi**on** (GRIPHON) trial, the administration of selexipag reduced the risk of death or complications related to PAH by 40%, compared to placebo.^7^ Overall, 14.3% of patients in the selexipag group and 7.1% of patients in the placebo group discontinued their study regimen prematurely because of adverse events. Recent data on selexipag use in real-world settings in the US and Europe are available, but limited information exists on patient persistence to selexipag in Canada, and data on predictors of persistence are sparse.^8^^,^^9^ In a retrospective, observational study from the US, of 3931 patients who reached a maintenance dose of selexipag, 68.2% were persistent at 6 months, and 48.4% were persistent at 12 months.^10^ However, this study did not include Canadian patients or investigate the impact of any factors as potential predictors of persistence.

The primary objectives of this study were to estimate real-world persistence on selexipag in Canadian patients with PAH and identify potential predictors of selexipag persistence.

## Methods

### Study design

**Figure 1:**
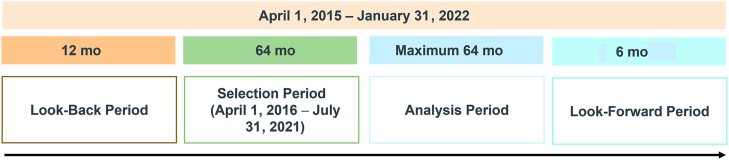
Study periods.

This retrospective claims database analysis included adult (aged ≥ 18 years) PAH patients with ≥ 1 selexipag claim from the Ontario Drug Benefits (ODB), Régie de l'assurance maladie du Québec (RAMQ), or Canadian Private Drug Plan (PDP) databases, between April 2016 and July 2021 (Fig. 1). These claims databases include longitudinal, anonymized, individual-level reimbursement data for retail pharmaceutical claims. The PDP data reflect national coverage for private payers, capturing approximately 80% of all pay-direct private plan claims across Canada, whereas data from the ODB and the RAMQ captured 100% and 50% of Ontario and Quebec public drug plan claims for the PAH market, respectively. Only 50% of the RAMQ data was available based on the data-sharing agreement with RAMQ as part of a privacy protection policy to provide a random sample of patients. The database captures patient demographics (age, sex, province), postal code of pharmacy (ODB and PDP only), dispensing date, drug name, days of supply, drug cost, and prescriber specialty (where available). The dataset does not include diagnostic codes; therefore, PAH and comorbidities were inferred based on medication claims (Supplemental Table S1). The final protocol was submitted to a central ethics committee (Advarra) with a request for exemption from institutional review board oversight. The request was sought because this study was retrospective and longitudinal and used de-identified claims data that posed no risk to the included patients.

### Study population

At least one selexipag claim in the selection period was used to infer a diagnosis of PAH, as use of selexipag is not approved or reimbursed in Canada for other any indications. Patients were included if they had at least 1 year of history in their current insurance plan. Patients who were not active in the drug plan in the 1 year prior to the index date, which was defined as the first day of selexipag prescription, were excluded.

### Measurements

Baseline patient characteristics and history of medications were recorded in the 1-year look-back period prior to the index date. Comorbidities were inferred for diabetes, heart failure, chronic kidney disease, chronic obstructive pulmonary disease or asthma, and pulmonary fibrosis, using claim history for medications listed in the Supplemental Table S1 6 months prior to the index date. The use of the PAH medications epoprostenol, treprostinil, bosentan, ambrisentan, macitentan, tadalafil, sildenafil, and riociguat was recorded. Use of diuretics (any) and anticoagulants (any) in the 1 year prior to the index date was also recorded. Use of specific combinations of PAH medications in the 6 months prior to the index date was recorded as double oral combinations (ERA + PDE5i or ERA + riociguat) and other combination (ERA + PDE5i + parental prostacyclin).

### Follow-up

Patients were followed in the claims database for a maximum of 64 months from the index date until they were administratively censored or had discontinued selexipag. All patients had a 6-month look-forward period after the end of their follow-up period to assess activity in their insurance plan. Patients were considered to have discontinued selexipag if they either switched to parenteral prostacyclin analogue or did not have a claim indicating renewal of their selexipag prescription within 60 days from when their prior prescription should have been completed. This 60-day grace period was used to allow for real-world disruptions (for example, hospitalizations) to continuous claims in patient insurance plans. Patients were censored administratively if they did not have any additional claims for selexipag after a 60-day grace period and were not active in their insurance plan after their last recorded selexipag prescription, or if they reached the end of the study period. Patient persistence on selexipag was calculated as the number of months from the index selexipag claim in the insurance plan to the earliest estimated date of discontinuation, censoring, or the end of the analysis period. Within the databases used, the reasons for discontinuation or censoring, including death, are not available.

### Statistical analysis

Kaplan-Meier estimates were used to describe proportions of patients persistent on selexipag during the follow-up period. Among the available variables, initial predictor selection for selexipag persistence was based on frequency, distribution, missingness of available candidate variables, and inputs from clinical experts. Variable importance in predicting discontinuation was evaluated using machine-learning models for analysis of right-censored survival data. The modelling analysis used the following 4 feature-selection models: stepwise Cox proportion hazards (PH), Boruta, Coxnet, and Random Survival Forest. A total of 28 predictors were analyzed in each model. Features included patient demographic information, baseline medications, and inferred health conditions (full list in Supplemental Table S2).

To assess the impact of a lack of physician familiarity with selexipag as a potential factor in prescription and persistence, the time from selexipag reimbursement date to the index date was calculated for all patients that indexed after the date of reimbursement for their respective insurance plans. For patients in PDP, the notice of compliance date (market authorization by Health Canada) for Canada was used (January 20, 2016). For patients in the ODB and RAMQ, the respective provincial dates of public reimbursement were used (June 14, 2018 and March 3, 2018, respectively). To assess the potential impact of the COVID-19 pandemic on prescription patterns, patients were flagged if their index date was between March 1, 2020 and the end of the study period in July 2021. Insurance type was also included as a model feature and categorized as private (PDP) and public (aggregated ODB and RAMQ).

Features included in the 4 models were ranked per the individual model and then given an average rank across the 4 models. Instead of using the feature importance of a single model, the average was taken to increase our confidence that a variable was truly important, regardless of the model used to derive its importance. This approach helps ensure that if a single model had very extreme results compared to the others, they could be averaged out across multiple models.

The 12 most important features based on average rank were built into a Cox PH model to assess the impact of these predictors on patient persistence with selexipag, as previously described.^11^ Persistence predictors were reported as hazard ratios (HRs) with associated *P*-values and 95% confidence intervals (CIs). SAS version 9.4 (SAS Institute, Cary, NC) was used for data extraction from the database, data manipulation, and data analysis. Descriptive statistics were reported as mean, standard deviation, median, interquartile range, and number and percentage for categorical variables. Missing data were not imputed but were reported. All values with a count of < 6 patients or claims were suppressed according to privacy rules. Secondary suppression of other cells was also done to avoid back calculation of small cells. Percentages were not calculated for suppressed cells.

## Results

### Patients’ characteristics

**Figure 2:**
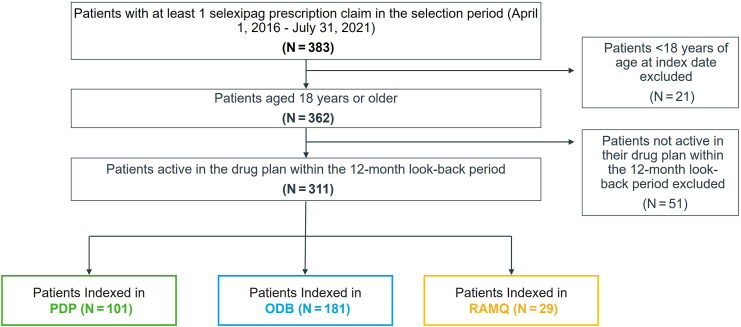
Patients with pulmonary arterial hypertension included in each of the Claims databases. ODB, Ontario Drug Benefits; PDP, Private Drug Plan; RAMQ, Régie de l’assurance maladie du Québec.

A total of 311 patients were included in the study, with 101 (32%) in PDP, 181 (58%) in ODB, and 29 (9%) in RAMQ (Fig. 2). Patient characteristics are shown in Table 1. Most patients were female, and aged between 50 and 79 years. In PDP, most patients were from Ontario (n = 58; 57%) and Quebec (n = 31; 31%). Total numbers of patients from other provinces were suppressed due to small cell sizes (count < 6 patients). Most patients in PDP (92%) and ODB (78%) had their first selexipag claim filled at a pharmacy in an urban area.

**Table 1:** Baseline pulmonary arterial hypertension medications and comorbidities reported by database (N = 311)

Demographics	PDP (N = 101)	ODB (N = 181)	RAMQ (N = 29)	Total (N = 311)
Age categories, y				
18–34	3∗	13 (7)	3∗	21 (7)
35–49	24 (24)	24∗	3∗	51 (16)
50–64	59 (58)	35 (19)	10 (34)	104 (33)
65–79	8 (8)	97 (54)	13 (45)	118 (38)
≥ 80	3∗	11∗	3∗	17 (5)
Female sex	68 (67)	132 (73)	18 (62)	218 (70)
Time from market authorization to index date, y, mean (SD)	2.8 (1.6)	1.5 (1.0)	1.5 (0.9)	2.0 (1.4)
Medications†				
Bosentan	13∗	52 (29)	3∗	68 (22)
Ambrisentan	11∗	38 (21)	3∗	52 (17)
Macitentan	45 (45)	3∗	11∗	59 (19)
Epoprostenol	3∗	3∗	3∗	3∗
Treprostinil	3∗	3∗	3∗	3∗
Riociguat	6∗	3∗	0 (0)	9 (2.9)
Sildenafil	9 (8.9)	15 (8.3)	0 (0)	24 (7.7)
Tadalafil	60 (59)	137 (76)	23 (79)	220 (71)
Diuretics	56 (55)	143 (79)	16 (55)	215 (69)
Anticoagulants	29∗	45 (25)	3∗	77 (25)
Inferred comorbidities‡				
Diabetes	13∗	43 (24)	3∗	59 (19)
Heart failure	59 (58)	142 (78)	20 (69)	221 (71)
Chronic obstructive pulmonary disease	24 (24)	67 (37)	13 (45)	104 (33)
Pulmonary fibrosis	3∗	0 (0)	0 (0)	3∗

Values are n (%), unless otherwise indicated.

ODB, Ontario Drug Benefits; PDP, Canadian Private Drug Plan; RAMQ, Régie de l'assurance maladie du Québe; c SD, standard deviation.

∗ All values with a count of < 6 patients or claims were masked as 3∗ according to privacy rules. Secondary suppression was used to avoid back calculation of a small cell. Percentages were not calculated for suppressed cells.

† Medications in the 6 months prior to index date.

‡ Comorbidities were inferred based on medications.

**Figure 3:**
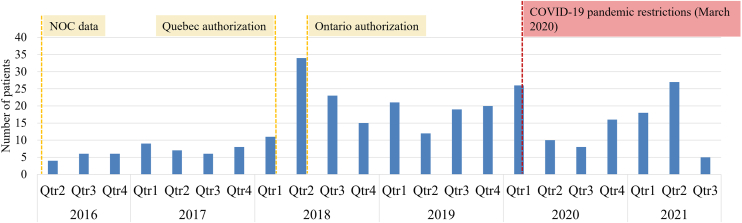
Distribution of patient index dates for all databases, NOC, notice of compliance; ODB, Ontario Drug Benefits; PDP, Private Drug Plan; Qtr, quarter; RAMQ, Régie de l’assurance maladie du Québec, 2016–2021 (N = 311).

Patient assistance programs to support access to selexipag were in place after the notice of compliance in Canada, and therefore, 36 patients (12%) had an index date prior to public reimbursement and are still recorded in the claims databases. A total of 275 patients (88%) had an index date after the date of reimbursement authorization for their insurance type (public vs private), with a mean (standard deviation) time from reimbursement to index date of 2.0 (1.4) years. The number of patients indexed numerically increased following public reimbursement by the RAMQ and ODB (Fig. 3).

In the 6 months prior to the index date, a majority of patients were on PDE5i (n = 244; 78%) and ERAs (n = 173; 56%), whereas 9 patients (2.9%) were on riociguat. Approximately one third of patients were on double oral combination therapy (n = 99; 32%). At the index date, 115 patients (37%) received selexipag as part of triple oral combination therapy, and 63% received it as dual therapy.

Concomitant diuretics were frequent (n = 215; 69%). The most common inferred comorbidities among patients with PAH were heart failure (n = 221; 71%), chronic obstructive pulmonary disease or asthma (n = 104; 33%), and diabetes (n = 59; 19%).

### Persistence on selexipag

**Figure 4:**
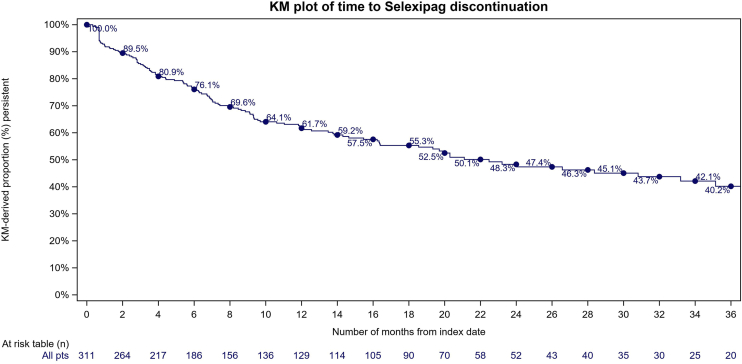
Kaplan-Meier (KM) estimates of persistence on selexipag for patients (pts) with pulmonary arterial hypertension in the claims database (N = 311).

A total of 129 patients (41%) discontinued selexipag in the study period, including 16 (5%) that switched to parenteral prostacyclin analogues. Almost one quarter of patients (n = 69; 22%) were censored during the study period, and 113 (36%) were still persistent on selexipag at the end of the study period. The estimated persistence on selexipag at 6, 12, 24, and 36 months was 76.1%, 61.7%, 48.3%, and 40.2%, respectively (Fig. 4).

The 12 features included as predictors in the final model (in order of average model rank, from 1 to 12) are presented in Table 2, with a high hazard ratio (HR) indicating a higher likelihood of discontinuation of selexipag, and a lower HR indicating a higher likelihood of selexipag persistence. No predictors in the final model were significant (*P* < 0.05), but a history of claims for double oral combination was associated with a trend for enhanced persistence (HR 0.63; 95% CI 0.38-1.04, *P* = 0.075), whereas baseline sildenafil (HR 1.92; 95% CI 0.97-3.58, *P* = 0.051) and riociguat (HR 2.31; 95% CI 0.89-5.23, *P* = 0.06) tended to be associated with lower persistence on selexipag.

**Table 2:** Hazard ratios for selexipag discontinuation in the final Cox proportional hazards model

Feature	Final rank	HR (95% CI)∗	*P*
Double oral combination (baseline)	1	0.63 (0.38–1.04)	0.075
Triple oral combination (baseline)	2	0.77 (0.48–1.23)	0.28
Sildenafil (baseline)	3	1.92 (0.97–3.58)	0.051
Index selexipag days supply	4	1.00 (0.97–1.02)	0.74
Index selexipag dosage, mg†	5	1.00 (0.99–1.01)	0.65
Diabetes (baseline)	6	0.71 (0.41–1.18)	0.21
Age‡, y	7		
≥ 80		Reference	
65–79		0.63 (0.32–1.35)	0.20
50–64		0.58 (0.29–1.28)	0.15
35–49		0.70 (0.31–1.63)	0.38
20–34		0.78 (0.29–2.08)	0.61
Diuretics (baseline)	8	1.39 (0.89–2.21)	0.16
Index selexipag cost	9	1.0 (1.0-1.0)	0.52
Riociguat (baseline)	10	2.31 (0.89–5.23)	0.06
Time since market authorization (index)	11	1.06 (0.92–1.22)	0.45
Tadalafil (baseline)	12	1.04 (0.66–1.64)	0.87

CI, confidence interval; HR, hazard ratio.

∗ A higher HR indicates a higher likelihood of discontinuation of selexipag; a lower HR indicates a higher likelihood of selexipag persistence.

† Total dosage (mg) of the index selexipag claim.

‡ A final rank was assigned to the predictor “age group,” (in years) which had 4 categories: (20–34, 35–49, 50–64, and 65–79) presented separately.

## Discussion

This study provides real-world evidence focusing on selexipag persistence in a sample of the Canadian PAH population. The main findings of this claim analysis are as follows: (i) estimated selexipag persistence was 40.2% at 36 months, with a short median persistence for the overall cohort of approximately 22 months; and (ii) no independent predictors of selexipag persistence were identified.

The low persistence with selexipag (40.2%) by 36 months has several potential explanations. First, known side effects may limit the tolerability of therapies targeting the prostacyclin pathway. Even in a more selected population and the stringent context of a clinical trial, selexipag was discontinued by 14% of patients in the phase 3 GRIPHON trial. Adherence is almost always lower in real-world data, compared to that in trial populations.^12^ Secondly, although selexipag reduces the risk of clinical worsening, patients with PAH may still deteriorate despite its addition, which may result in discontinuation of selexipag and escalation to a parenteral prostanoid, such as intravenous epoprostenol. Lastly, although mortality data were not available in these databases, PAH carries a high annual risk of death, and some of these patients likely succumbed to the disease during the observation period. Overall persistence with selexipag at 6 and 12 months was 76.1% and 61.7%, respectively. This rate was higher, compared to 68.2% and 48.4% for the same time points from one US observational study.^10^

This study from the US only calculated selexipag discontinuation among patients who had reached a maintenance dose of selexipag. Of the patients that met an individualized maintenance dose in that population, 32.8% were considered to be on a medium dose (600-1000 μg), and 52.4% were on a high dose (1200-1600 μg). In contrast, persistence in this claims analysis was calculated in a cohort of patients from their index (first) selexipag claim in the insurance plan; therefore, patients from the titration phase were included. Another recent real-world study from the US found no difference in time to discontinuation of selexipag among patients on a low-, medium-, or high-maintenance dose.^13^ Compared to other classes of PAH therapies, such as ERAs, the persistence with selexipag is similar. For example, a study from Japan found that the mean persistence with ERAs ranged from 10-18 months,^14^ and a US study reported that the median persistence with ERAs ranges from 12.6-15.8 months.^15^

No independent predictors of selexipag persistence were identified in this study. Factors that may impact patient persistence, generally, include side effects, barriers to accessing PAH treatment (including limited access to a specialist or availability of the drug at pharmacies), increasing treatment complexity, management of treatment interruptions, and presence of comorbidities.^16^^,^^17^ Some of these factors may be less relevant in the Canadian context in which PAH therapies are prescribed only by PAH experts in regional referral centres and typically are dispensed via specialty pharmacies. Several factors in the analysis (eg, sildenafil, riociguat use) trended toward statistical significance, and the study may have been underpowered to detect a true association. Also, other complex factors that were unmeasured in this study may drive selexipag tolerability, adherence, and persistence. Although adherence to medication differs from persistence, a point of interest is that other studies have found few predictors of adherence to PAH medications, other than frequency of dosing, time from diagnosis, and need for a copay.^18^, ^19^, ^20^ Future studies from patient registries or administrative datasets are thus needed to explore more clinically focused variables to refine the predictors of persistence. Poor patient persistence on treatment can be of serious concern in chronic diseases such as PAH and may impact the real-world rates of mortality, despite the presence of effective treatment options. Although the results of the model may provide some direction into medications or demographic characteristics that may be relevant to selexipag persistence, no clear predictors of persistence in this population emerged from the features included in the model.

### Limitations

These claims database analyses have several limitations. First, medications dispensed to patients in hospitals are not captured in this database. Given that PAH is a severe and progressive disease, hospitalizations may occur for short periods of time in which a patient will not have any claims for selexipag using their insurance plan. To account for this possibility, a grace period of 60 days was used to increase the specificity of patients being classified as discontinued or censored. Nonetheless, the cohort of patients who discontinued in this analysis may include patients with therapy gaps (including long hospitals stays) of more than 60 days following their last prescriptions’ days supply. However, if patients initiate selexipag in the hospital, their first claim would not be captured by the claims database, and persistence could be underestimated by the months missed at the beginning of treatment. Moreover, the cohort in this analysis had geographic representation across Canada for patients with private insurance, but data on patients with PAH with public insurance outside of Ontario and Quebec were not available. This lack of data may impact the generalizability of these data in provinces where information was not available. In addition, patients that are censored in this study include those that have no further claims in any therapeutic markets after their final selexipag claim (and grace period), which could be the result of interprovincial migration, emigration, loss of insurance coverage, or switching from private to public plans. Similarly, as the claims database does not include patient outcome data, patients that die during the follow-up period are included in the cohort of administratively censored patients.

The claims database does not have any diagnostic information or associated diagnostic codes (eg, International Classification of Disease codes). Therefore, all diagnoses of PAH or any of the comorbidities of interest were inferred based on the medication claim history, which could result in misclassification of patients as either having or not having one of the diseases of interest. This risk is mitigated in this study, as selexipag is only indicated for PAH in the Canadian market. One possibility is that off-label use of selexipag in patients with non-group 1 pulmonary hypertension occurred during the study period, but we assumed this occurred very infrequently during the early years of its availability in Canada. Inferred diagnoses of other comorbidities of interest, however, may be over- or under-estimated depending on the sensitivity and specificity of the medication algorithm. For instance, the incidence of heart failure is likely overestimated, as diuretics may be used specifically for PAH, and this medication is included in the algorithm for inferring heart failure (Supplemental Table S1). Finally, estimates of persistence must be interpreted carefully, as the claims database can only describe patterns of pharmaceutical claims, not patient compliance or adherence to the medication. This analysis assumes that patients with continuous selexipag claims are taking the medication as prescribed. The reasons for discontinuation are also unknown.

## Conclusion

Real-world data from the Canadian population suggest a level of persistence on selexipag that is comparable to that for other PAH medications. Further research into predictors of selexipag persistence is needed to identify reasons for discontinuation, to help optimize use of this medication in real-world populations, which could help improve treatment outcomes.

## Footnotes

To access the supplementary material accompanying this article, visit *CJC Open* at https://www.cjcopen.ca/ and at https://doi.org/10.1016/j.cjco.2025.10.009.
