# Supplementary material for: Real-world Persistence on Selexipag for Pulmonary Arterial Hypertension in Canada
Source: CJC Open. 2025 Oct 25;8(2):231–8. doi: 10.1016/j.cjco.2025.10.009 (PMC12946791; doi:10.1016/j.cjco.2025.10.009)
Supplement: Supplemental Materials [file mmc1.docx]

**Supplemental Materials**

**Supplemental Table S1.** Drugs used to infer co-morbidities

| Inferred Co-morbidity | Drug Grouping | | | |
| --- | --- | --- | --- | --- |
| Heart Failure | ACE | ARB | Beta Blocker | Diuretics |
|  | Entresto | Lancora | MRA | SGLT2 inhibitors |
| Chronic Obstructive Pulmonary Disease or Asthma | Advair | Combivent Respimat | Other Bronchial | Spiriva |
|  | Alvesco | Daxas | Other ICS | Spiriva Respimat |
|  | Anoro Ellipta | Duaklir Genuair | Oxeze | Symbicort |
|  | Arnuity Ellipta | Flovent | Pulmicort | Tudorza Genuair |
|  | Atrovent Mdi | Incruse Ellipta | Saba | Ultibro Breezhaler |
|  | Breo Ellipta | Inspiolto Respimat | Seebri Breezhaler | Zenhale |
|  | Combivent Mdi | Onbrez Breezhaler | Serevent |  |
| Pulmonary Fibrosis | Ofev | Esbriet |  |  |
| Diabetes | Avandamet | Forxiga Franchise | Invokana Franchise | Starlix |
|  | Avandaryl | Glimepiride | Januvia Franchise | Tolbutamide |
|  | Avandia | Glp-1 | Jardiance Franchise | Trajenta Franchise |
|  | Bydureon | Glucobay | Metformin | Trulicity |
|  | Byetta | Glumetza | Nesina Franchise | Victoza |
|  | Chlorpropamide | Glyburide | Onglyza Franchise |  |
|  | Diamicron & Generics | Glyxambi | Pioglitazone |  |
|  | Diamicron Mr & Generics | Insulin | Repaglinide |  |
| Chronic Kidney Disease | Lanthanum Carbonate | Magnesium Carbonate | Paricalcitol | Sevelamer Carbonate |
|  | Sevelamer Hydrochloride | Sucroferric Oxyhydroxide |  |  |

**Supplemental Table S2.** Features for selexipag persistence model selection

| Category | Predictor level | Potential predictors in model |
| --- | --- | --- |
| Demographics | N/A | • Age group  • Sex  • Province  • Forward sortation area (FSA)  • Type of insurance coverage |
| Selexipag prescription | Per claim | • Prescription days supply  • Prescription cost  • Index prescription dosage |
| Prescriptions for PAH-related medications (baseline) | Medication | • Bosentan  • Ambrisentan  • Epoprostenol  • Macitentan  • Riociguat  • Sildenafil  • Tadalafil  • Treprostinil  • IV Prostacyclin |
| Combination therapy for PAH (baseline) | Combination of medications | • Double Oral Combination = ERA + PDE5i or ERA + Riociguat  • Triple Oral Combination = ERA + PDE5i + Selexipag  • Other Triple therapy = ERA + PDE5i + IV Prostacyclin |
| Medications claimed for other conditions (baseline) | Indication | • Type 2 diabetes  • Heart failure  • Chronic Kidney Disease  • Chronic obstructive pulmonary disease (COPD)  • Pulmonary fibrosis interstitial lung disease (ILD) |
| Patient index relative to time from selexipag market authorization | N/A | Time from selexipag market authorization for patient insurance type (Year) |
| Patient index relative to date of COVID-19 restrictions | N/A | Patient entire follow-up period post-pandemic restrictions in March 2020 |
